# Supplementary material for: Oxidative stress and associated clinical manifestations in malaria and sickle cell (HbSS) comorbidity
Source: PLoS One. 2022 Jun 8;17(6):e0269720. doi: 10.1371/journal.pone.0269720 (PMC9176834; doi:10.1371/journal.pone.0269720)
Supplement: Attachment — Submitted filename: Review PLOS_PONE-D-21-30010.pdf [file pone.0269720.s003.pdf]

PONE-D-21-30010

The present study proposes to carry out a study that investigates the impact of condition of having concomitant sickle cell disease and malaria, comparing with individuals with malaria and with sickle cell disease, describing biomarkers associated with renal, hematological, and oxidative stress changes and clinical manifestation.

The authors should include in their Introduction, more about SCD pathophysiology, and about the hemolytic aspect of the disease, that is hereditary; also, and about epidemiology of malaria in the region. What kind of Plasmodium is common in the region? The methodology is well described but will be important place about how the hospital work, and how many patients of each disease they receive. The authors include Bilirubin as a renal marker, it is necessary to explain it, because there is a mistake about this data. About the ethics aspect, as they included patients over 10 years old, it will be important to place the parental consent and that the Declaration of Helsinki was followed. The authors need to place clearly that comorbidity is related to the presence of malaria and SCD.

In the results, authors should include more specific data, and confirm data about age in the table 1 and in the text. In table 2, will be important to include how many patients they found in each group of parasite density sub-range. The results are well presented, but need to correct some mistake, such as, the authors referrer leukocytes as level and not count.

The discussion needs to be rewritten the discussion, and please they need to include more about the hemolysis marker investigated at the study, there are several markers, but the authors need to justify their choice. It is important because, there is a paper that reports about the influence of hemolysis in the 8-iso-prostaglandin F2α levels, that need to be incorporated at the discussion section (Ulrike Dreiβigacker et al. Clinical Biochemistry 43 (2010) 159–167). When the authors talk about the “EBR cut-off value greater than 10 could be used to predict malaria in SCD”, there is a several important aspects that need to be incorporate such as the SCD crisis.
