# Supplementary material for: Oxidative stress and associated clinical manifestations in malaria and sickle cell (HbSS) comorbidity
Source: PLoS One. 2022 Jun 8;17(6):e0269720. doi: 10.1371/journal.pone.0269720 (PMC9176834; doi:10.1371/journal.pone.0269720)
Supplement: Attachment — Submitted filename: Review Comments Plos 1.docx [file pone.0269720.s002.docx]

**Review Comments**

I would like to congratulate the authors for taking this personal initiative to understand the clinical manifestations in these disease conditions. However, I have enumerated a few points for the authors to address and upgrade the manuscript.

1)Was the study both a retrospective and prospective Cohort study given that data was collected from people already suffering from SCD coming to the clinic for visits and people diagnosed with malaria and SCD.

2)Under study design you wrote its a cross-sectional study, could you please state the appropriate study design

3)Given that Malaria infectivity varies seasonally, were the samples collected at same peak seasonal period or randomly? This is to minimize variations in samples parameters

4)In the discussion section, please elaborate more on the leukocyte ratio effect on the disease condition results

5)Was informed consent obtained before engaging the patients in the study. If yes please clearly state that and precise the age groups from which consent was obtained from

6) You stated in the manuscript that `the oxidative stress status of sickle cell patients with malaria has not been studied’. Provide more clarity on this e.g see publication below

Atiku SM, Louise N, Kasozi DM. Severe oxidative stress in sickle cell disease patients with uncomplicated Plasmodium falciparum malaria in Kampala, Uganda. BMC Infect Dis. 2019;19(1):600. Published 2019 Jul 9. doi:10.1186/s12879-019-4221-y
