# Supplementary material for: Oxidative stress and associated clinical manifestations in malaria and sickle cell (HbSS) comorbidity
Source: PLoS One. 2022 Jun 8;17(6):e0269720. doi: 10.1371/journal.pone.0269720 (PMC9176834; doi:10.1371/journal.pone.0269720)
Supplement: Attachment — Submitted filename: Response to Reviewers.docx [file pone.0269720.s005.docx]

Reviewer #2:

Comment: The authors carefully answered all comments made by the reviewers. The manuscript has improved considerably, with its purpose clearly stated and brings important contributions on the occurrence of malaria in individuals with sickle cell anemia. However, after reading all suggestions and recommendations, and also all results presented, this reviewer consider that the conclusion of the study, both in the abstract and in conclusion section, should be very well evaluated, once it is placed “Exogenous antioxidant supplement is suggested for sickle cell patients with malaria to neutralize the increasing levels of free radicals which are known to have deleterious consequences on cells and organs biomolecules”, since the data presented do not allow the authors to carry out the recommendation that the simple presence of the investigated marker, the isoprostane, 8-isoprostaglandin F2α (8-iso-PGF2α) serving as a basis to stimulate the use of external antioxidant agents, since the authors did not carry out functional studies that support the indication; therefore, the suggestion of this reviewer is that this recommendation should be withdrawn from the conclusion of and also from the abstract, since other markers of oxidative stress were not studied and functional studies with antioxidant therapeutic agents were not performed in this study, in a way that confirms the possibility of using these agents or specific agents in the treatment of individuals with sickle cell disease and malaria.

Response:

We can confirm that the said recommendation has been expunged from the manuscript
